# Ginsenosides Rb1 Attenuates Chronic Social Defeat Stress-Induced Depressive Behavior *via* Regulation of SIRT1-NLRP3/Nrf2 Pathways

**DOI:** 10.3389/fnut.2022.868833

**Published:** 2022-05-12

**Authors:** Ning Jiang, Yiwen Zhang, Caihong Yao, Hong Huang, Qiong Wang, Shuangxue Huang, Qinghu He, Xinmin Liu

**Affiliations:** ^1^Research Center for Pharmacology and Toxicology, Institute of Medicinal Plant Development, Chinese Academy of Medical Sciences, Peking Union Medical College, Beijing, China; ^2^Hunan University of Chinese Medicine, College of Traditional Chinese Medicine, Changsha, China; ^3^Affiliated TCM Hospital, School of Pharmacy, Sino-Portugal TCM International Cooperation Center, Southwest Medical University, Luzhou, China

**Keywords:** ginsenoside Rb1, depression, CSDS, mice, neuroinflammation

## Abstract

Ginsenoside Rb1, a diol-type ginseng saponin, has various positive effects on the central nervous system. This study aimed to evaluate the antidepressant effects of Rb1 on chronic social defeat stress (CSDS) induced behavioral deficits and the exact neural cascades linked with inflammatory processes. The results of behavioral tests such as social interaction, tail suspension, and forced swimming revealed that oral treatment of Rb1 (35 and 70 mg/kg) alleviates depression-like behavior. Rb1 treatment increased antioxidant enzyme activity (SOD and CAT) and reduced lipid peroxidation (LPO) content in the hippocampus. Rb1 also suppressed the production of inflammatory cytokines (TNF-α, IL-18, and IL-1β) as well as microglial activation (Iba1) in response to CSDS. Moreover, Rb1 administration considerably reduced the protein expression of NLRP3 (inflammasome) and promoted the protein expressions of Nrf2, HO-1 and Sirtuin1(SIRT1) activation in the hippocampus. Our findings showed that Rb1 effectively restores the depressive-like behavior in CSDS-induced model mice, mediated in part by the normalization of oxidative stress levels. The suppression of neuroinflammation is mediated by the regulation of SIRT1-NLRP3/Nrf2 pathways. Our results asserted that the Rb1 is a novel therapeutic candidate for treating depression.

## Introduction

Depression is an affective mental disorder, mainly characterized by significant and persistent depression, anhedonia, hopelessness, and worthlessness, accompanied by a syndrome of neurotic dysfunction (1). According to the World Health Organization (WHO), in 2008, depression ranked as the third most severe complication. It will rise to first place by 2030. Furthermore, the high rate of suicide and self-injury of depression seriously harms patients' physical and mental health, work, study, life, and social interaction and causes great social and financial load (2, 3). Even though a variety of antidepressants are now available to treat depression, the first choice drugs, such as selective 5-HT reuptake inhibitors (SSRIs), have a minimal potential therapeutic ratio, a long list of adverse effects, poorer clinical compliance, and a high rate of recurrence after drug withdrawal (4). Hence, developing candidate therapeutic approaches with greater potency and comparatively less side effects are vital to prevent and treat depression.

Depression's pathogenesis is extremely complex and enigmatic. Multiple studies have reported that neuro-inflammation is due to the elevated pro-inflammatory cytokines in the brain. In recent years it has been considered a key factor in developing depression (5). Clinical studies demonstrated increased pro-inflammatory cytokine levels in the peripheral circulation and some brain regions in patients with depression (6). In animal models, recent findings have shown that inflammation plays a role in the progression of depressive and anxiety-like behavior in response to stress stimuli such as chronic unpredictable mild stress and social defeat stress (CSDS) (7).

NLRP3 inflammasome is a multimeric protein complex containing cytosolic NLRP3, the adaptor protein ASC, and Caspase-1 that triggers innate immune responses plays an important role in the maturation and release of inflammatory factors (8). The NLRP3 inflammasome is activated by numerous divergent invading pathogens and cellular damages such as ROS, mitochondrial DNA, ATP, and subsequent excretion of pro-inflammatory cytokines such as IL-18 and IL-1β into the extracellular matrix and prolonged immunological reactions that finally result in neurotransmitter dysfunction, oxidative damage to neurons, etc. (9). The NLRP3 inflammasome was found to be increased in the depression patient's peripheral blood mononuclear cells (PBMCs) (10). In animal models of depression, the depression-like phenotype is accompanied by NLRP3 inflammasome activation in the brain. Additionally, numerous pieces of evidence suggest a significant association between the NLRP3 inflammasome activation in the stress responses and depression's underlying causes, and inhibition of it exerts as a promoting therapeutic target for depressive disorders (11, 12).

Several studies have shown that increased reactive oxygen species (ROS) overcome the antioxidant defense system, resulting in oxidative stress, which plays a role in the pathophysiology of various disorders, including depression (13). ROS plays a crucial role in activating inflammasome, and pretreatment with various ROS scavengers represses NLRP3 inflammasome activation in response to a series of agonists (14, 15). The major transcriptional factor governing inflammation and oxidative stress in depression is nuclear factor E2-related factor 2 (Nrf2) (16). Under oxidative stress, Nrf2 is transported to the nucleus and binds to antioxidant response elements (ARE) to regulate the production of antioxidant enzymes such as HO-1 (17). The Nrf2/HO-1 signaling pathway plays an important role in anti-inflammatory activities. The upregulation of Nrf2/HO-1 and downregulation of IL-1β, IL-6, TNF-α in microglia have improved depressive-like behavior (18, 19). Furthermore, recent research has shown that Nrf2 regulates NLRP3 activation in LPS-induced depression, and the Nrf2/NLRP3 pathway is closely linked to the development of depression (20, 21).

Sirtuin1(SIRT1) is a NAD-dependent histone deacetylase belonging to the class III histone deacetylase family. SIRT1 is widely distributed in the brain. High levels in the hippocampus and cortex play a pivotal role in cellular events such as aging, inflammation, homeostasis, metabolic activities, and cellular survival (22). SIRT1 has recently been linked to major depressive disorder (23). Several studies in animal models also support the important role of SIRT1 in preventing and treating depression. Some previous studies have demonstrated that inflammatory cytokine expression is inhibited by SIRT1 via mediating initiation and progression of inflammation (e.g., deacetylating NF-kB) and ultimately preventing behavioral deficits (depressive and anxiety disorders) caused by chronic stress in rodents (24, 25). A recent study reported that an elevated expression of SIRT1 overcomes LPS-associated acute depressive-like behavior via suppressing microglial NLRP3 multiprotein oligomers known as Inflammasome (20).

Ginsenoside Rb1 (diol-type ginseng saponins) is considered the main active component of *Panax ginseng*. *Panax ginseng* is a traditional herb native to China, Siberia, and Korea. This herb has been widely used as a tonic for more than 2000 years in Far East countries. In Southeast Asian countries, it is used as a traditional remedy for preventing and treating neuropsychiatric diseases, such as depression (26). Our recent work showed that Rb1 can significantly ameliorate depressive-like behavior induced by chronic social defeat stress and modulates the BDNF-Trkb signaling pathway (27). Ginsenoside Rb1 demonstrated substantial antidepressant effects in rats by modulation of amino-acidergic and monoaminergic receptors and their associated neurotransmitters, induced microglial activation, and improved adult hippocampal neurogenesis in mice subjected to chronic mild stress (28). Unfortunately, very little is known about Rb1's antidepressant-like effect on behavioral impairments caused by CSDS, or on the exact neuronal processes underlying, or about Rb1's significance to the neuroprotective effect of slowing the inflammatory process in depression. Given these facts, the existing study aimed to examine the antidepressant effect of Rb1 in CSDS-associated depression-like behavior by assessing the behavioral changes and relations between neuroinflammatory parameters, such as microglia activation and the NLRP3 signaling pathway, as well as oxidative stress and the Nrf2/HO-1 signaling pathway in the hippocampus. Moreover, the expression of hippocampal SIRT1 was also estimated for unraveling possible mechanisms.

## Materials and Methods

### Materials

Ginsenoside Rb1 (Rb1, purity > 98%) was provided by Chengdu Ruifensi Biological Technology Co., Ltd, China. Imipramine (IMI) was acquired from Sigma-Aldrich USA).

### Animals

**Figure 1:**
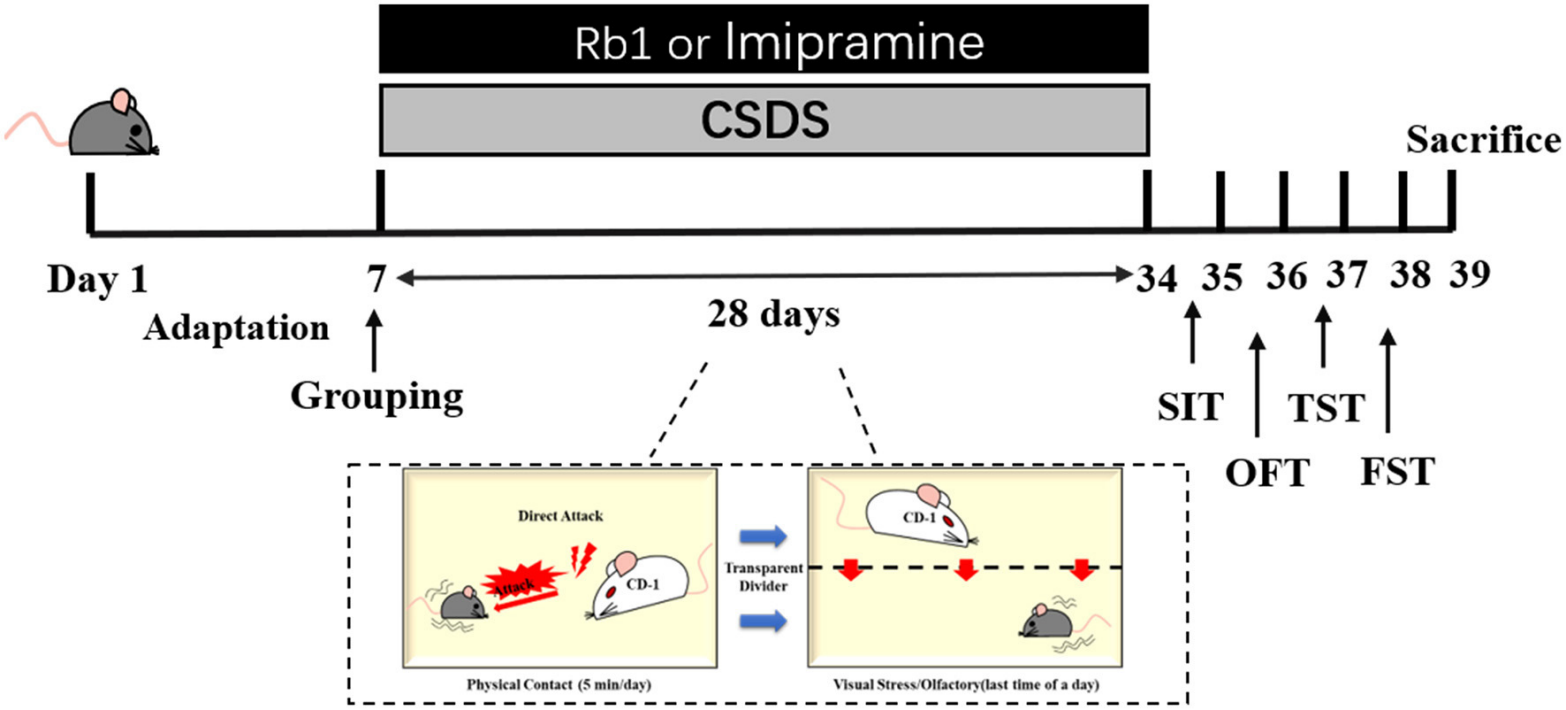
Illustrates the experimental protocol utilized in the present study.

Vital River Co., Ltd. (Certificate No. SCXK 2016–0006, Beijing, China) supplied 6-8 weeks old male C57BL/6J mice (*n* = 60, weight: 20-22 g); and 12-months old retired breeders male CD-1 mice (*n* = 70, weight: 20-22 g). All mice were housed in standard animal housing conditions, including a humidity of 55%, a 20-22°C temperature, a 12-h:12 h light/dark cycle, and unlimited access to water and food. The animal experiments were performed with proper approval (Approval No. SYXK 2017-0020) and agreed with the requirements outlined by the Animal Research Committee of Peking Union Medical College's Institute of Medicinal Plant Development. After 1 week of adaptation, the male mice were randomly divided into control, model, and treatment groups, i.p., imipramine (15 mg/kg), and Rb1 (35 and 70 mg/kg) (27). Rb1 and imipramine were suspended in distilled water using ultrasound. Except for the control group, the animals were subjected to a CSDS treatment followed by a series of behavioral assessments over the next four weeks. Imipramine (15 mg/kg) and Rb1 were administered orally for 32 days (Figure 1).

### CSDS Procedure

The CSDS mouse model was developed according to the previously reported procedure with slight changes (7, 29, 30). Shortly after an intrusion into their cage, CD1 mice were used to observe the aggressive behavior of CSDS-induced mice (31). C57BL/6 mice were physically defeated for 28 days by being exposed to aggressive CD1 mice for 5 min each day. The C57BL/6 mice were placed in the same cage as the aggressor mouse the next day, isolated by a clear porous organic acrylic plate (thickness = 4 mm), and subjected to continuous psychological stress for the next 24 h, including frightening auditory, olfactory, and visual stimuli. Porous transparent organic acrylic sheets were placed to isolate the control mice in the same cage.

### Behavioral Testing

#### Social Interaction Test

As previously reported, the **Social Interaction Test** (SIT) was carried out with minor modifications. A two-stage SIT was utilized to assess social avoidance behavior, and a video recording was made by 2-stage SIT (32). Each C57 mouse was caged in the arena and permitted to move freely for 150 s without a CD1 aggressor mouse in the interaction zone (IZ) in the first stage. The mice were taken out of the arena for 30 s at the end of the first phase, followed by cleaning the arena. After that, a CD1 mouse was caged using a transparent plastic box and released into the arena with the test mouse. The second step was then carried out for 150 s, and the same metrics were recorded again. Time spent in the IZ, both with and without the target, has been followed.

#### Open Field Test

On day 36, a computer-aided recorder was employed to monitor the mice's locomotor activity (LMA) using the open field test. A video recorder was placed on the top of a 75 cm diameter, 40 cm high metal container. Four mice were caged in the center of each box and permitted to roam free for 5 min for every test.

#### Tail Suspension Test

As per prior reports, mice were used in the tail suspension experiment (33, 34). Adhesive tape (about 1 cm from the tail tip) was used to suspend the mouse for 6 min. Following 2 min habituation, the immobility time was recorded during the final 4 min using Tail Suspension Real-Time Analysis (System-2.0).

#### Forced Swimming Test

The FST test was performed according to the earlier reported procedure (35). At about 25°C, the mice were placed in an acrylic tube (diameter = 14 cm and a height = 20 cm) confined to a depth of 15 cm for 6 min. For the last 4 min of a 6 min examination, Tail Suspension Real-Time Analysis (System-2.0) was used to take a video of immobility time.

### Measurement of the Oxidative Stress and Inflammatory Factors

The hippocampus were homogenized with cold saline (10 volumes). The Pierce BCA Assay kits were used to determine protein concentration, while BSA was used as a reference. The CAT and SOD activity and LPO level in the hippocampus of mice were measured and estimated using commercial assay kits (Jiancheng Biology, China), following manufacturer instructions. One unit of SOD activity was defined as the amount that reduced the absorbance by 50% at 450 nm. The CAT activity was calculated according to the amount of the yellow complex produced by the reaction between H2O2 and ammonium molybdate at 405 nm. LPO were measured using the thiobarbituric acid reactive substance (TBARS) method as previously described in 535 nm. The adduct was maximally absorbed at a wave length of 586 nm. The LPO content and SOD and CAT activities in serum are expressed as μmol/l, U/ml, and U/ml, respectively (36). The levels of proinflammatory mediators, such as tumor necrosis factor α (TNF-α), interleukin 18 (IL-18), and interleukin 1β (IL-1β), in the serum were determined with commercial enzyme-linked immunosorbent assay (ELISA) kits for mice (Clound-clone, China) according to the manufacturer's instructions.

### Western Blotting

Western blotting was performed with minor modifications as previously described (37). Following the approved operating conditions, cells were separated and loaded onto Millipore PFDF membranes (Bedford, MA, USA). After 2 h of blocking in 5% nonfat milk in Tris-buffered saline with Tween-20 (TBST), the membranes were treated with the primary antibodies described below overnight at 4°C: SIRT1 (ab189494, 1:1,000), NLRP3 (Q8R488, 1:1,000), cleaved Caspase-1 (89332s, 1:1,000), ASC (ab180799, 1:1,000), IL-1β (YT5201, 1:1,000), HO-1 (43966s, 1:1,000), Nrf2 (ab137550, 1:1,000), β-actin (4967s, 1:1,000). The membranes were treated for another 1 h at room temperature with a horseradish peroxidase-conjugated secondary antibody. ECL Prime Kit was used to visualize the protein bands, and ImageJ 1.46r software (NIH, USA, RRID: SCR_003070) was utilized to quantify them.

### Immunohistochemistry

Following the behavioral tests, the animals were sedated with pentobarbital sodium and then transcranial perfused with 4% paraformaldehyde in 0.01 M phosphate buffer for 24 h after the last session. All the immunohistochemistry procedures were conducted according to our previous method (38). Every Iba-1^+^ cell within the DG was counted, and each group's fluorescence intensity was evaluated through ImageJ software (Media Cybernetics, USA). A total of five mice were selected per group for the assessment.

### Statistical Analysis

The SPSS Statistics version 21.0 (Chicago, USA) was employed to analyze the obtained results. The differences in the mean values were calculated using one-way ANOVA. A significant difference (LSD) test was employed for one-way ANOVA analyses, followed by a *post-hoc* test. The obtained results were indicated as the mean ± SEM. The *p*-value <0.05 was regarded as statistically significant.

## Result

### Impact of Rb1 on Depressive-Like Behavior in CSDS Mice

**Figure 2:**
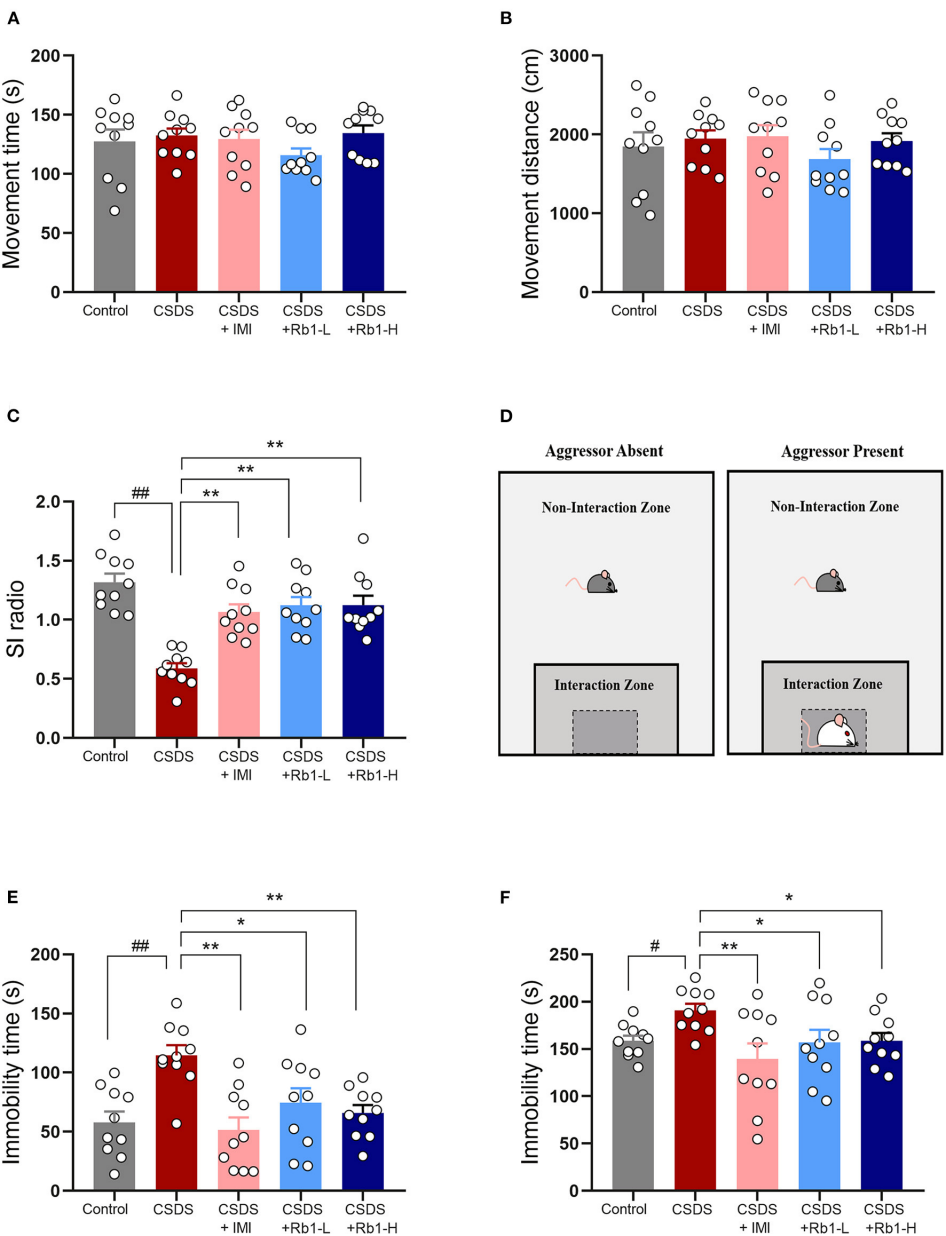
The Rb1 treatment enhanced CSDS-induced depression-like behavior. **(A,B)** Open field test (OFT); **(C)** Social interaction test (SIT); **(D)** Aggressor approach—social interaction test paradigm; **(E)** Tail suspension test (TST); **(F)** Forced swim test (FST). The entire data is presented and articulated as means ± SEM; *N* = 10 mice per group. ^#^*p, and* ^*##*^*p* < 0.05, and 0.01, substantially varied from control; ^*^p and ^**^p < 0.05, and 0.01, significantly differed from CSDS group.

There were no significant variations in the locomotor activities of all groups in the OFT, as indicated in Figures 2A,B. The underlined data suggested that the locomotor activities were unaffected by the drug or the CSDS treatment. According to the SIT, after 4 weeks of CSDS treatment, the social interaction ratio in CSDS mice was lowered than in the control mice (*p* < 0.01; Figures 2C,D). Rb1 treatment (35 and 70 mg/kg) or positive control drug imipramine (15 mg/kg) considerably reverse the decrease after 28 days of treatment (all *p* < 0.01). In TST and FST, the CSDS model group had a significantly longer immobility time, as shown in Figures 2E,F (*p* < 0.01). Rb1 (35 and 70 mg/kg) or imipramine (15 mg/kg) administration completely abolished the increase in immobility time induced by CSDS (*p* < 0.05).

### Rb1 Attenuates the Inflammatory Cytokines Production and Oxidative Stress in the Hippocampus of CSDS Mice

**Figure 3:**
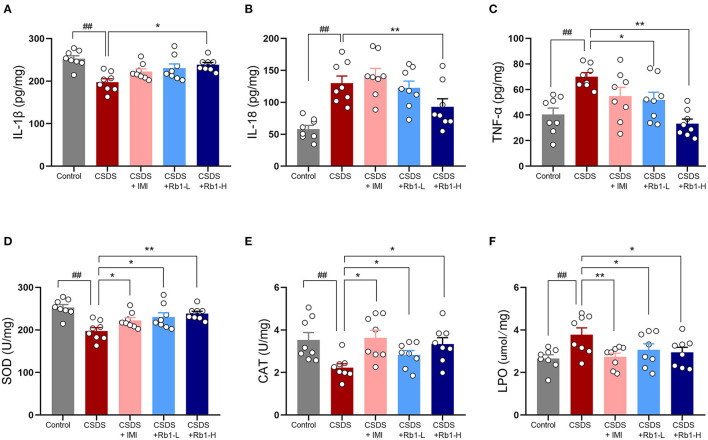
Ginsenosides Rb1 and their effects on inflammatory and oxidative stress markers in CSDS mice; **(A)** Expression of IL-1β; **(B)** Expression of IL-6; **(C)** Expression of TNF-α; **(D)** SOD activity; **(E)** CAT-related activities; **(F)** Activity at the LPO level in the hippocampus. The entire data has been presented are means ± SEM; *N* = 10 mice per group. ^*##*^*p* < 0.01, considerably differed from control; ^*^p and ^**^p < 0.05, and 0.01, significantly varied from CSDS group.

The hippocampus of the CSDS model group showed a substantial elevation in hippocampal inflammatory markers such as TNF-α, IL-18, and IL-1β (Figures 3A-C; *p* < 0.01). Rb1 therapy, on the other hand, considerably reduced the CSDS-induced elevation in the underlined markers (*p* < 0.05). Relative to the control group, the CSDS group had noticeably lowered SOD and CAT activity in the hippocampus but greatly increased LPO levels (Figures 3D-F; all *p* < 0.01). Rb1 (35 and 70 mg/kg) or imipramine (15 mg/kg) therapy, on the other hand, increased SOD and CAT activity whilst substantially lowering the high content of LPO in the hippocampus (*p* < 0.05).

### Effects of Rb1 on the SIRT1 Expression in the Hippocampus in CSDS Mice

**Figure 4:**
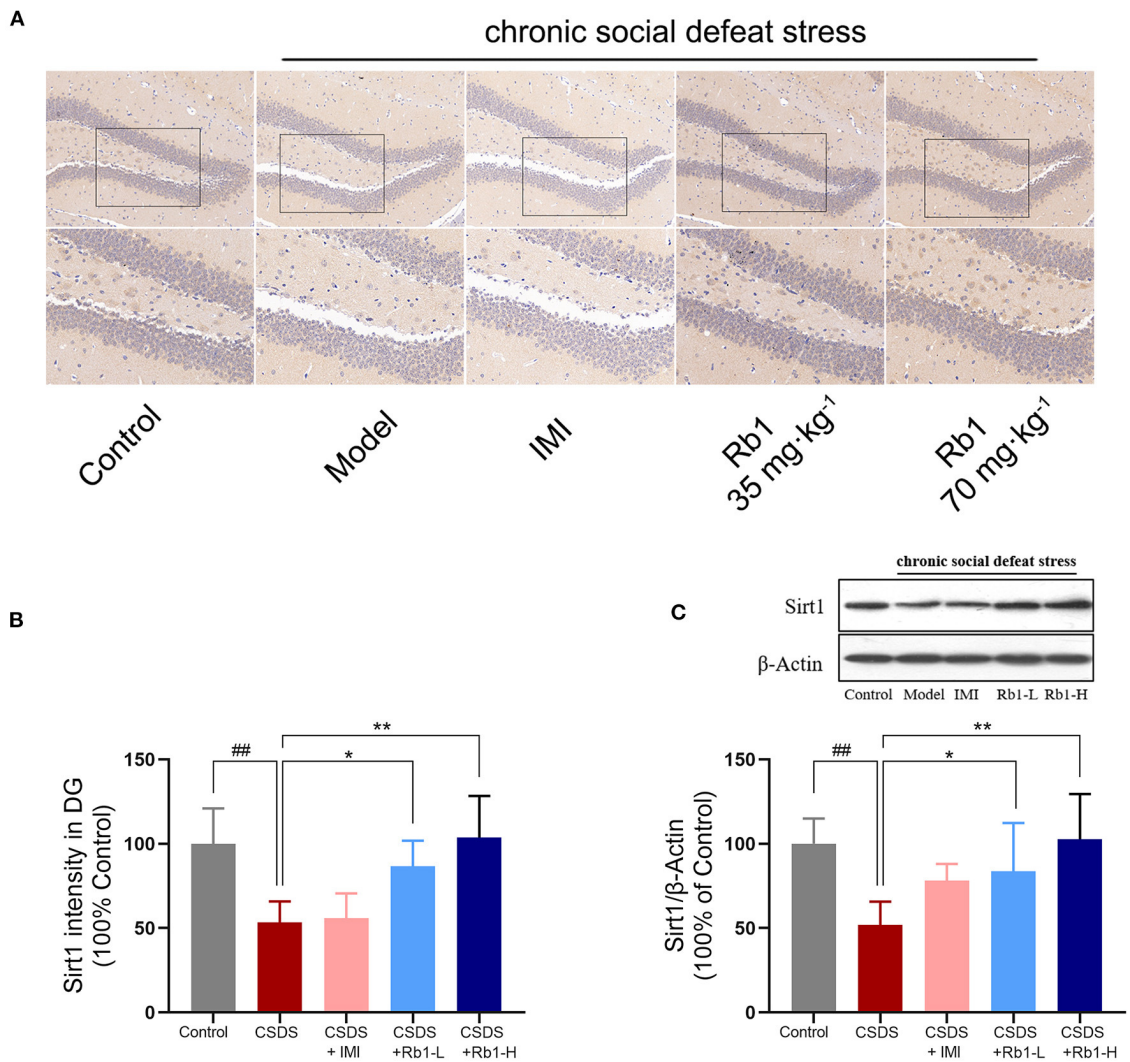
In CSDS mice, ginsenosides Rb1 increased SIRT1 expression in the hippocampus. **(A)** SIRT1 expression in the rat hippocampal DG area, as seen by IHC staining. region (200, bar = 100 μm; 400, bar = 50 μm); **(B)** The mean fluorescence intensities of SIRT1 in the DG region; **(C)** Western blotting for detecting the SIRT1 expression in the hippocampus. The entire data has been presented are means ± SEM; *N* = 4 mice per group. ^*##*^*p* < 0.01, considerably varied from control; ^*^p and ^**^p < 0.05, and 0.01, considerably varied from CSDS group.

IHC results showed that SIRT1 expression in the DG regions was decreased following a 28-day CSDS, while this decrease was reversed by Rb1 treatment (Figure 4A). The model group's relative fluorescence intensity in the DG areas was dramatically reduced, as seen in Figure 4B (*p* < 0.01). Conversely, Rb1 (35 and 70 mg/kg) exposure significantly increased the relative fluorescence intensity of SIRT1in the CSDS mice (*p* < 0.05, *p* < 0.01). Furthermore, Western blotting revealed that the amount of SIRT1protein was found to be considerably lowered in the hippocampus of mice (exposed to CSDS) than that of control mice (Figure 4C; *p* < 0.01). However, the Rb1 therapy prevented the lowered expression of SIRT1.

### Rb1 Alleviates NLRP3 Inflammasome and Reduces Microglial Stimulation in the Hippocampus in CSDS Mice

**Figure 5:**
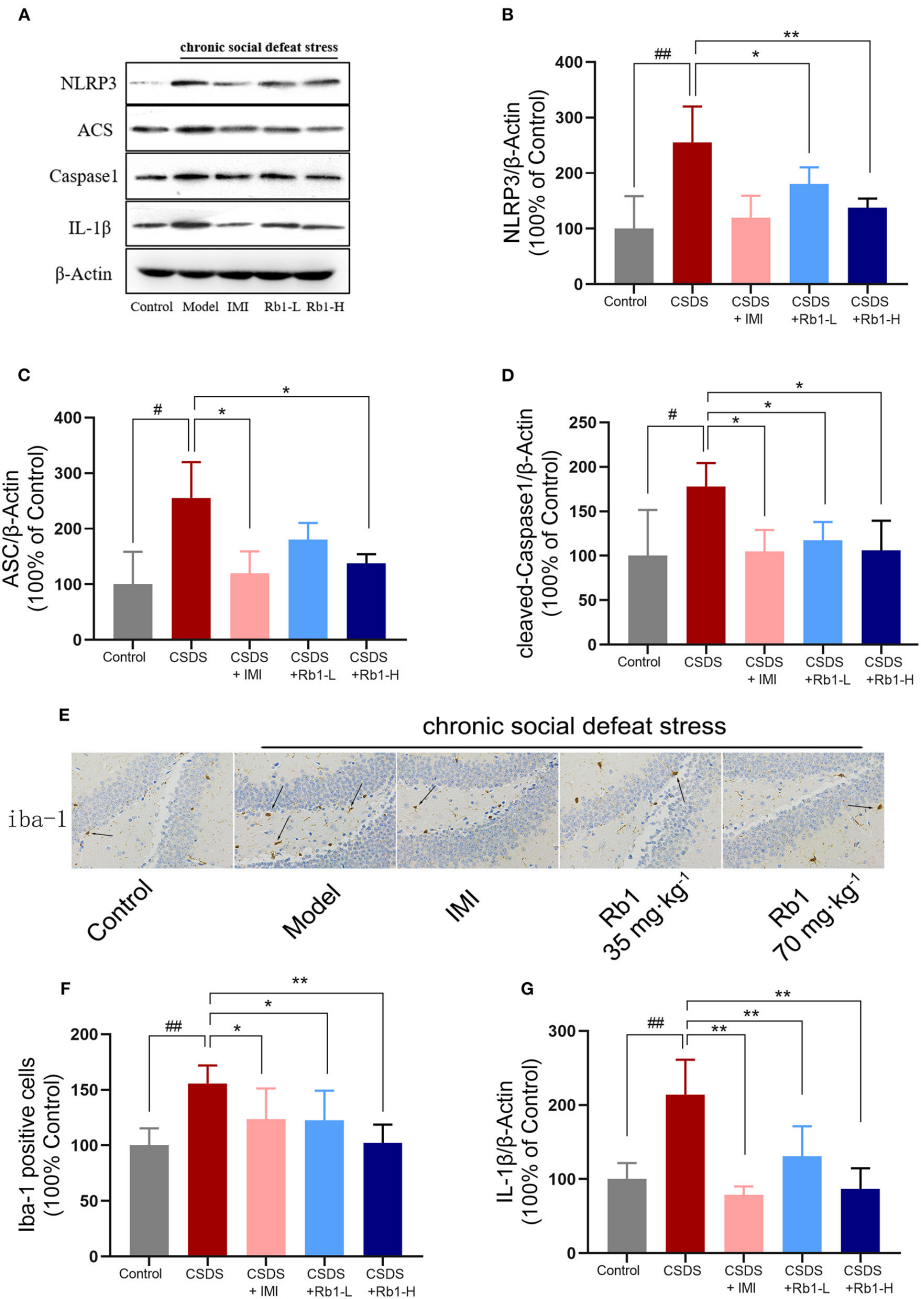
In the hippocampus of CSDS mice, ginsenosides Rb1 reduced the NLRP3 inflammasome and microglial activation. Western blots analysis demonstrates **(A)** results of protein expression in the hippocampus of CSDS mice; **(B)** NLRP3; **(C)** ASC; **(D)** Cleaved Caspase-1; **(G)** IL-1β; **(E)** Immunohistochemical staining of Iba-1 in the hippocampus of CSDS mice (×400); **(F)** The number of Iba-1 positive cells. The entire data has been presented are means ± SEM; *N* = 4 mice per group. ^#^*p*, and ^*##*^*p* < 0.05, and 0.01, significantly different from control; ^*^p and ^**^p < 0.05, and 0.01, considerably varied from CSDS group.

The hippocampal expressions of cleaved Caspase-1, NLRP3, ASC, and IL-1β were considerably higher in the CSDS-exposed group than in the control group (Figures 5A–D,G; *p* < 0.05). In contrast, the expression of these proteins was dramatically reduced after treatment with Rb1. Immunohistochemistry for Iba1 revealed obvious alterations in the morphology of microglia in the dentate gyrus (DG) following CSDS. Iba1-positive microglia in the DG of non-stressed mice possessed fewer and scattered processes, while CSDS-treated mice had larger cell bodies with thick and condensed processes (Figures 5E,F). Relative to the other groups, the number of Iba1-immunoreactive cells in the DG elevated substantially post CSDS procedure (Figure 5G; *p* < 0.01). Rb1 inhibited microglia over-activation by suppressing the translational level of ASC-1, NLRP3, and cleaved caspase-1.

### Impact of Rb1 on the HO-1 and Nrf2 Protein Expression

**Figure 6:**
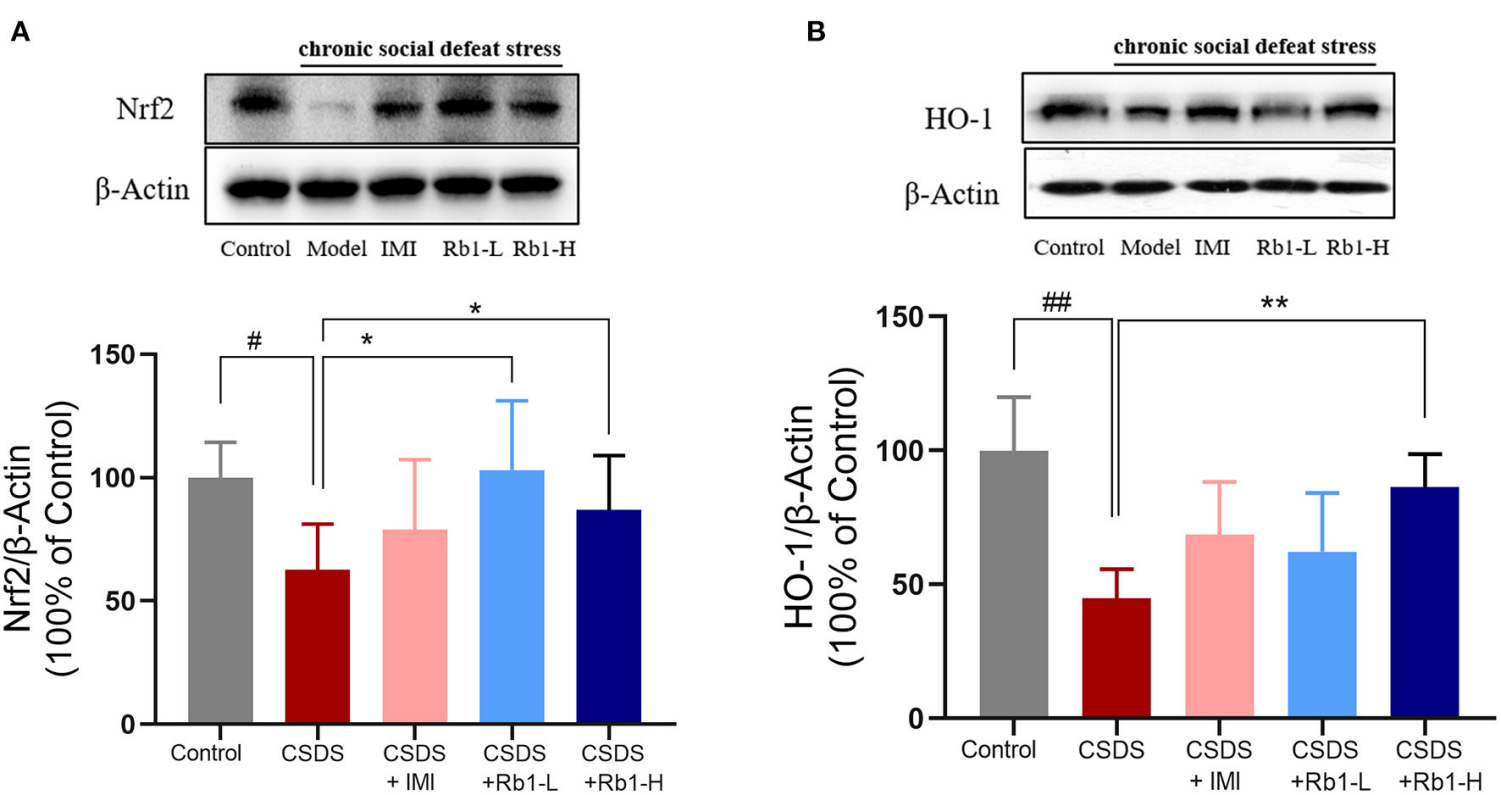
Ginsenosides Rb1 induced NRF2 and HO-1 upregulation in the hippocampus of CSDS mice. Western blots analysis illustrates **(A)** NRF2; **(B)** HO-1. The entire data has been presented are means ± SEM; *N* = 4 mice/group. ^#^*p*, and ^*##*^*p* < 0.05, and 0.01, accordingly, considerably different from control; ^*^p, and ^**^p < 0.05, and 0.01, significantly varied from CSDS group.

The expression of Nrf2 and HO-1 was substantially decreased in CSDS rats' hippocampus compared to the control rats (Figures 6A,B; *p* < 0.01). However, Rb1 (35 and 70 mg/kg) considerably reversed the decrease in the HO-1 expression (*p* < 0.05). Rb1 therapy at 70 mg/kg led to a significant elevation in the expression of Nrf2.

## Discussion

In the current investigation, it has been revealed that Rb1 improves the behavioral deficit in CSDS mice, suggesting that it has antidepressant properties. In the hippocampus, the CSDS-induced neuroinflammatory response and oxidative stress were both ameliorated by Rb1 administration. Furthermore, Rb1 treatment considerably reduced the translational level of NLRP3 (inflammasome) and triggered translational levels of Nrf2, HO-1, and SIRT1 activation.

The CSDS depression model is frequently used and shown to be helpful in the evaluation of chronic stress-related depression (39). In the current study, we discovered that 4-week CSDS exposure significantly caused a persistent set of depression-like phenotypes such as despair and social-avoidance behaviors, as shown by deficits in SIT but prolonged immobility time in TST and FST, as indicated earlier (40, 41). More importantly, we observed that Rb1 at doses of 35 and 70 mg/kg (ip) in mice produced a significant increase of social interaction ratio in SIT and reduction of immobility time in the TST and FST. At the same time, Rb1 treatment did not lead to any significant abnormalities in locomotor activity even at the higher dose (70 mg/kg), indicating that this compound was well tolerated. The improvement of these behavioral deficits was not due to locomotor abnormality. Our observations were tested using standard behavioral tests, which quantitatively supported our findings and showed consistency with present findings. These results imply that ginsenoside Rb1 has antidepressant-like properties in CSDS depressed mice.

A strong relationship between neuroinflammation and depression has been revealed in recent decades. Inflammatory responses associated with central and peripheral pro-inflammatory cytokines secreting microglia (activation) and ROS and RNS generation are triggered by chronic stress and eventually result in depression (42). Microglia are significantly stimulated in the CNS and peripheral blood of animal models of depression such as CSDS and CUMS, and pro-inflammatory cytokines including TNF-α, IL-1 β, and IL-6 are also significantly elevated in the brain, according to a preclinical study (43). In agreement, we found that CSDS significantly promoted the Iba-1 activation and increased the levels of TNF-α IL-1 βin in the hippocampus of mice. At the same time, Rb1 administration could reverse these effects that have been considerably associated with the attenuation of microglia activation and an elevation of pro-inflammatory cytokines.

Rb1 therapy had an inhibitory impact on NLRP3 inflammasome stimulation in CSDS mice, according to our findings. NLRP3 inflammasome is a key constituent of the innate immune response. It consists of multiple proteins, including NLRP3 (nucleoside-bound oligomeric nod-like receptor), ASC (apoptosis-related spotted protein), and caspase-1 (cysteine aspartic protease) (44). Activation of NLRP3 inflammasome mainly occurs in macrophages and microglia. The activated NLRP3 inflammasome causes the hydrolysis of the inactive pro-caspase-1 protein, which is then cleaved into active caspase-1 that converts IL-1β and IL-18 precursor proteins into mature IL-1β and IL-18 (45). The underlined signals are received by other associated cells, resulting in the amplification of the signal cascade, which finally leads to pathogenic alterations involved in depression. Microglia positive expression level and pro-inflammatory factor expression level are increased under chronic stress, and NLRP3-inflammasome inhibitor can inhibit chronic stress-induced behavioral abnormalities (46). Numerous studies have linked chronic social defeat stress-induced depression to an NLRP3 inflammasome-dependent inflammatory response in mice, as well as the suppression of the NLRP3 inflammasome by long-term antidepressant medication treatment (47). In this study, it has been revealed that the translational level of NLPR3, ASC, and cleaved caspase1 was considerably increased in CSDS mice, which was associated with increased production of inflammatory mediators and microglial activation. The underlined data showed consistency with the earlier research. However, Rb1 exposure considerably reduced the NLRP3-mediated inflammatory response and restored depression-like behavioral responses in CSDS animals. Previous studies reported that Rb1 inhibited inflammation and improved insulin signaling in adipose tissue by suppressing endoplasmic reticulum (ER) stress-associated NLRP3 inflammation activation (48). As a result, ginsenoside Rb1's putative antidepressant-like mechanisms may be linked to its positive effect in inflammation (CSDS-induced) in the hippocampus, which arises through suppressing of NLRP3 inflammasome stimulation.

Under normal settings, the dynamic balance between ROS and antioxidants is maintained; nevertheless, when the body is stressed, hyperactivation of active oxygen free radicals occurs, resulting in an imbalance of an organism's oxidation system. These effects cause mitochondrial damage, elevated levels of NO, MDA, and cytokine, a decrease in antioxidant activity (such as SOD, CAT, and GSH), hippocampus neuronal damage, apoptotic process, and depression, or worsening of the depression course (49, 50). Herein, MDA levels in the hippocampus of CSDS mice were found considerably higher, while CAT, SOD, and GSH levels were affectedly lowered. The underlined data showed consistency with the earlier studies. In contrast, Rb1 significantly increased the expression of antioxidant enzymes while reducing the content of the metabolite of lipid peroxidation (LPO), indicating a protective effect of Rb1 against oxidative stress impairment.

Furthermore, dysregulated redox-sensitive signaling plays a significant role in the immunological imbalance that often occurs with depression (51). An essential antioxidant cascade is Nrf2/HO-1, which contributes to maintaining redox balance and reducing oxidative damage by stimulating the expression of downstream antioxidant enzymes indicated as a candidate target for therapeutic approaches of depression (52). Depression-like symptoms can be alleviated by increasing the expression of Nrf2/HO-1 and decreasing the expression of IL-1β, IL-6, and TNF-α in the microglial cells. In addition, it has been postulated that CMS and CSDS depression models may reduce the expression of the Nrf2-system (Nrf2 and HO-1) (53). In agreement, our results of western blot revealed that the expression of Nrf2 and HO-1 were downregulated in the hippocampus of mice post 28 days of CSDS. Rb1 therapy, on the other hand, slightly improved the reduced expression of Nrf2 and its target proteins, such as HO-1. Ginsenoside Rb1 has been shown to have a neuroprotective impact by reducing oxidative stress in the CNS (54). Rb1 can enhance the expression of Nrf2 and HO-1 in the hippocampus of rats *in vivo* and *in vitro*. Its primary action mechanism is to activate Nrf2/HO-1, Nrf2/ARE signaling pathway, increase SOD and CAT activity, up-regulate endothelial nitric oxide synthase (eNOS), GSH, and HO-1 levels, reduce ROS and MDA content, thus improving intracellular redox status, reducing the expression of pro-apoptotic genes and inflammatory factor release in neuronal cells of rats, so as to alleviate damage of rat neuronal cells induced by oxidative stress (55, 56). These findings imply that Rb1 may stimulate the Nrf2/HO-1 signaling cascade, which in turn modulates the production of downstream antioxidants, potentially contributing to its antidepressant-like effects. These findings supported the use of Rb1 to reduce oxidative stress in CSDS-induced depressive behavior.

Moreover, we found that Rb1 treatment could upregulate the expression of SIRT1 in the hippocampus of depressive mice (CSDS-induced). Sirtuin 1 (SIRT1) has been linked to oxidative stress in neuroinflammation is involved in aberrant mood behavior in response to stressful situations like depression (25). SIRT1 can regulate several transcription factors, including NF-κB and TNF-α, and have a key role in regulating inflammatory processes and oxidative stress (57). Furthermore, SIRT1 is linked to the stimulation of the NLRP3 inflammasome (58). Salvianolic Acid B improved CUMS-induced depressive-like behavior by reducing the inflammatory process oxidative stress and stimulating the SIRT1 signaling cascade, according to Liao et al. (59). Rg1 exhibits antidepressant effects in CSDS mice via SIRT1 signaling cascades, according to prior research (7). In recent decades, it has been revealed that melatonin inhibits acute depressive-like behavior (LPS-induced) and microglial NLRP3 Inflammasome stimulation via the SIRT1/Nrf2 cascade. Here, our data showed that Rb1 significantly increased the expression of SIRT1. Meanwhile, the mediation effects of Rb1 were accompanied by the upregulation of Nrf2/HO-1 and downregulation of NLRP3 inflammasome, as well as the improvement in depressive-like behavior in mice (exposed to CSDS). It has been reported that ginsenoside Rb1 attenuates oxidative damage through the SIRT1 signaling pathways *in vitro* experiments (60). Ginsenoside Rb1 inhibited the expression of pro-inflammatory cytokines such as IL-1β, TNF-α, and IL-6 in I/R injury rats *via* activating of TLR4/MyD88 and SIRT1 signaling cascades (61). The underlined results revealed that Rb1 induced SIRT expression, which in turn regulates NLRP3 inflammasome activation and the Nrf2/HO-1 cascade and suppresses the elevated oxidative stress and inflammation, that results in reducing depressive-like behavior. Despite the interesting findings, limitations still exist in the current study. More studies are required to confirm whether Rb1 attenuates chronic social defeat stress-induced depressive behaviors through SIRT1 pathway by using SIRT1 inhibitor or knocking down Sirt1 *in vitro* and *in vivo* experiments.

In summary, our findings suggested that administering Rb1 can rescue the depressive-like behavior such as social avoidance and behavioral despair of the CSD-induced mice model. Rb1 attenuates pro-inflammatory cytokines production and inhibits the activation of NLRP3 inflammasome. Moreover, RbI normalized oxidative stress imbalance followed by the Nrf2/HO-1 and SIRT1 activation. The underlined findings shed light on the molecular mechanisms by which Rb1 attenuates neuroinflammation. Moreover, this study highlights Rb1 as a candidate novel therapeutic agent for the prevention and treatment of depression.

## Data Availability Statement

The original contributions presented in the study are included in the article/supplementary materials, further inquiries can be directed to the corresponding author/s.

## Ethics Statement

The animal experiments were carried out with proper approval (approval no. SYXK 2017-0020) and in agreement with the requirements outlined by the Animal Research Committee of Peking Union Medical College's Institute of Medicinal Plant Development.

## Author Contributions

NJ and XL designed the research. NJ, CY, HH, and YZ conducted the experiments. NJ, CY, and YZ performed the data analysis. NJ, YZ, QW, SH, QH, and XL wrote and amended the manuscript. XL and SH supervised the study and contributed to project administration. All authors approved the final version.

## Funding

This work was supported by the International Cooperative Project of Traditional Chinese Medicine (GZYYG2020023), the Innovation Fund for Medical Sciences (CIFMS) grant (2021-1-I2M-034) and the Space Medical Experiment Project of the China Manned Space Program (HYZHXM05003).

## Conflict of Interest

The authors declare that the research was conducted in the absence of any commercial or financial relationships that could be construed as a potential conflict of interest.

## Publisher's Note

All claims expressed in this article are solely those of the authors and do not necessarily represent those of their affiliated organizations, or those of the publisher, the editors and the reviewers. Any product that may be evaluated in this article, or claim that may be made by its manufacturer, is not guaranteed or endorsed by the publisher.
